# What you need to know about the world. Toward a taxonomy of planetary health knowledge

**DOI:** 10.3389/fpubh.2025.1564555

**Published:** 2025-05-09

**Authors:** Franziska Rees, Oliver Wilhelm

**Affiliations:** Department Individual Differences and Psychological Assessment, Institute of Psychology and Education, Ulm University, Ulm, Germany

**Keywords:** planetary health knowledge, taxonomy, jingle-jangle fallacies, sustainability, global citizenship, planetary health, knowledge

## Abstract

Knowledge about the world is deemed a core competence to engage in shifting the world to a better place to live. Although the importance of this knowledge is emphasized in several political and educational frameworks, there is still a lack of a definition of the scope of the required knowledge. Looking for a suitable taxonomy about world knowledge for sustainable development we analyzed different approaches in this area—Sustainable Development Goals, Global Citizenship, and Planetary Health—concluded that none of these approaches satisfied the requirements of a knowledge taxonomy and identified a huge amount of overlapping content among these approaches. By merging these frameworks, we developed the *Planetary Health Knowledge* (PHK) taxonomy targeting knowledge about the health of the planet, human individuals, human systems, and their interaction. This hierarchical taxonomy exhaustively and disjunctively covers all relevant aspects of PHK in seven domains: *Health, Nutrition, Environment, Safety, Education, Standard of Living*, and *Political and Economic Systems*. We further discuss the existence of *jingle-jangle fallacies* in this field, quality criteria for evaluating taxonomies, and possibilities to use the PHK taxonomy.

## 1 Introduction

Matters of fact are stubborn things. Knowledge about facts is often deemed essential for finding solutions to pervasive problems. Nowadays, we face numerous challenges such as climate change, poverty, hunger, and infectious diseases—broadly classified as environmental and societal challenges within the context of sustainable development. Knowledge about these global issues is deemed fundamental for tackling them (1–5). As such, it has been integrated in educational programs (2, 4, 6) and has even been a critical area of investigation in the recent OECD Programme for International Student Assessment (PISA) 2022 framework (7).

Although the critical role of knowledge about global issues has been emphasized by various experts (2–4), and the integration into educational frameworks, the definition of knowledge required to tackle global issues remains vague, and the specific interpretation of what belongs to the vaguely defined fields is up to the user (8). We assume that such knowledge is not exclusively to specific groups or experts but can be acquired regardless of ethnicity, nationality, gender, or other characteristics.

Focusing on knowledge, this contribution aims to bridge this definitional gap by providing a structured taxonomy of the required knowledge. More specifically, we develop a taxonomy of knowledge about the current status of the world, current salient world problems, their causes and corollaries, antecedents, and consequences, to which we refer as *Planetary Health Knowledge* (PHK).

### 1.1 Knowledge as a prerequisite to tackle global issues

Scholars, policymakers, and international organizations agree that knowledge about current global issues is a prerequisite to tackling them (2, 4). Without knowledgeable awareness of these problems, meaningful solutions remain inconceivable. Overarching frameworks such as Oxfam's (2) Global Citizenship Education framework, Planetary Health Education (6), Planetary Health Literacy (1), and Education for Sustainable Development (9) emphasize the role of knowledge, skills, and values in empowering individuals to take informed action.

Recent research found that informed citizenship (i.e., political knowledge) is associated with political participation and social and political engagement. Moreover, knowledge about ongoing political issues seems to be a key driver of active democratic participation and civic engagement (10). Evidently, it is impossible to be knowledgeable about all ongoing political processes, and therefore, being informed alone is unlikely to be a sufficient predictor for political and democratic participation (11). Instead of all individuals being fully informed, Moe (11) suggests that it is more important to distribute knowledge within social networks for political participation. It is a truism that humans cannot possibly know all facts, and knowledge about salient world problems is no exception to this rule. However, retrieving stored knowledge immensely facilitates the comprehension of novel information when contemplating problems, and remains the best predictor of further knowledge acquisition (12, 13).

Knowledge also provides a basis for pro-environmental behavior (14–16). However, the direct and indirect paths linking knowledge to behavior are somewhat complex and insufficiently understood. The strength of the association between knowledge and behavior might depend on the kind of knowledge targeted in tests. It is commonly assumed in applied psychology that declarative knowledge does not directly influence behavior, whereas action-related knowledge and effectiveness knowledge have a stronger impact on a person's behavior (14, 16). Yet, empirical analyses indicate that environmental knowledge is best represented by a general factor rather than three distinct types (15), suggesting that it reflects one overarching ability rather than three separate ones. Consequently, the assumption that different forms of knowledge vary significantly in their predictive power for behavior is questionable. Needless to say, knowledge is not nearly an exhaustive predictor of behavior. In applied social psychology, behavior is investigated as a function of knowledge, values, intentions, and attitudes (16–19). Accordingly, meaningful behavior interventions often incorporate knowledge components to enhance their effectiveness (20–22).

### 1.2 The need for a taxonomy of planetary health knowledge

Given the acknowledged importance of knowledge in addressing global challenges (1–5), there is a consequently growing demand for comprehensive knowledge tests as assessment tools to evaluate the understanding of the world's current state and salient global issues. Such tests require a well-defined and clearly structured taxonomy to systematically derive test content and ensure conceptual alignment. However, no such taxonomy defining the scope of this knowledge exists yet.

We define PHK as knowledge about the state of the world, salient global issues, their causes and consequences across three domains: The field of the health of the planet, the health of human beings, and the health of human (political, economic, and social) systems.

Existing knowledge tests in the field of PHK, such as world knowledge (23), sustainability knowledge (24), or environmental knowledge [e.g., (14–16, 25)] predominantly focus on environmental aspects or adopt broader frameworks such as the UN's Sustainable Development Goals (SDGs) (24, 26) or Global Citizenship Frameworks (23). However, they are often short, focus exclusively on the USA, or integrate many cultural aspects, without covering all dimensions and aspects of PHK. Therefore, developing a comprehensive PHK taxonomy is a necessary first step (current contribution) before developing a structured knowledge test [Rees and Wilhelm (Manuscript in preparation)]^1^.

In line with Revelle (27) and Saucier and Srivastava (28), a taxonomy should meet several criteria: i) Exhaustiveness—encompassing all relevant aspects of the domain, ii) Disjunction—ensuring that each component is uniquely categorized at any hierarchical level, iii) Usefulness—providing meaningful insights, practical applicability, and societal relevance, iv) Parsimoniousness—maintaining conceptual clarity by including only indispensable aspects, v) Replicability—ensuring for intersubjective dependable allocation to branches of the taxonomy. Meeting these criteria will not only facilitate the development of PHK tests but also advance our understanding of the role of knowledge in addressing planetary challenges.

### 1.3 Current study: purpose of the contribution

The primary objective of this contribution is to develop a comprehensive PHK taxonomy that systematically structures knowledge elements relevant to PHK based on the given definition of the field. To achieve this, we adopt a literature-based approach identifying and integrating existing frameworks that categorize pervasive world problems for policy interventions and public awareness.

Numerous concepts, frameworks, and branches of science have emerged in different disciplines in recent years, addressing issues related to the environment, human health, and social structures—independently or in interaction. Among these are: *Global Citizenship* (GC) (2, 4, 18), the SDGs (5), *Planetary Health* (PH) (29), One *Health* (OH) (30), *Doughnut Economics* (DE) (31). Out of these, we selected three frameworks—GC, SDGs, and PH—due to their broad scope and relevance. The SDGs provide the most comprehensive and structured categorization of global issues, covering environmental, health, and societal dimensions. We chose them over DE because they offer a more detailed and systematic framework, explicitly listing global challenges, whereas DE lacks this level of specificity. PH complements this by focusing on the interactions of human and planetary health, specifically addressing challenges due to global environmental changes. We chose PH over OH because of its broader scope. Last, GC offers an educational perspective focusing on characteristics of human beings.

We approach the taxonomy development in several steps. First, we briefly introduce the three selected frameworks (GC, SDGs, and PH) emphasizing their key elements and how they categorize global issues. This overview is not exhaustive and does not cover the full history or debates surrounding these frameworks. Rather, we focus on the relevant elements that address global challenges and evaluate each concept in terms of the criteria taxonomies should meet.

Analyzing these frameworks reveals substantial conceptual overlap, suggesting that many global issues are included in different approaches under different labels. This conceptual redundancy raises the issue of redundancy in the classification of global issues—a phenomenon discussed as *jangle fallacy* (32). This occurs when highly similar or identical concepts are labeled differently, which potentially hinders interdisciplinary collaboration. This challenge arises not least because the differing labels obscure the fact that the approaches actually address the same issues. Evidently, it might be considered a disservice to researchers but also policymakers, and citizens, that the same problem is addressed under different labels over and over. These jangle issues can complicate interdisciplinary collaboration, not least because, from looking at the labels and overall definitions, it is unclear, that all approaches actually address the same issues. It would be desirable but beyond the scope of this paper to clarify the potential uniqueness of each term in terms of terminological hygiene. We illustrate this redundancy identifying elements that appear multiple times across the concepts by bringing elements of the three approaches to a common language and assigning elements with similar content to each other, using the SDG goals as an anchor. Choosing the SDGs as anchor is not based on ideological preference, nor do we claim their superiority over other frameworks. Rather, the SDGs provide the largest, earliest, and most widely known collection of global issues, encompassing environmental, social, and economic dimensions.

After addressing this issue, we return to the PHK taxonomy development. Based on the extracted elements, we develop the PHK taxonomy by grouping the elements into thematically matching domains whilst keeping the domains as separate as possible (see 3.1 for details on the development process).

## 2 Analyzing existing frameworks

### 2.1 Introduction of approaches

#### 2.1.1 Global citizenship

**Figure 1:**
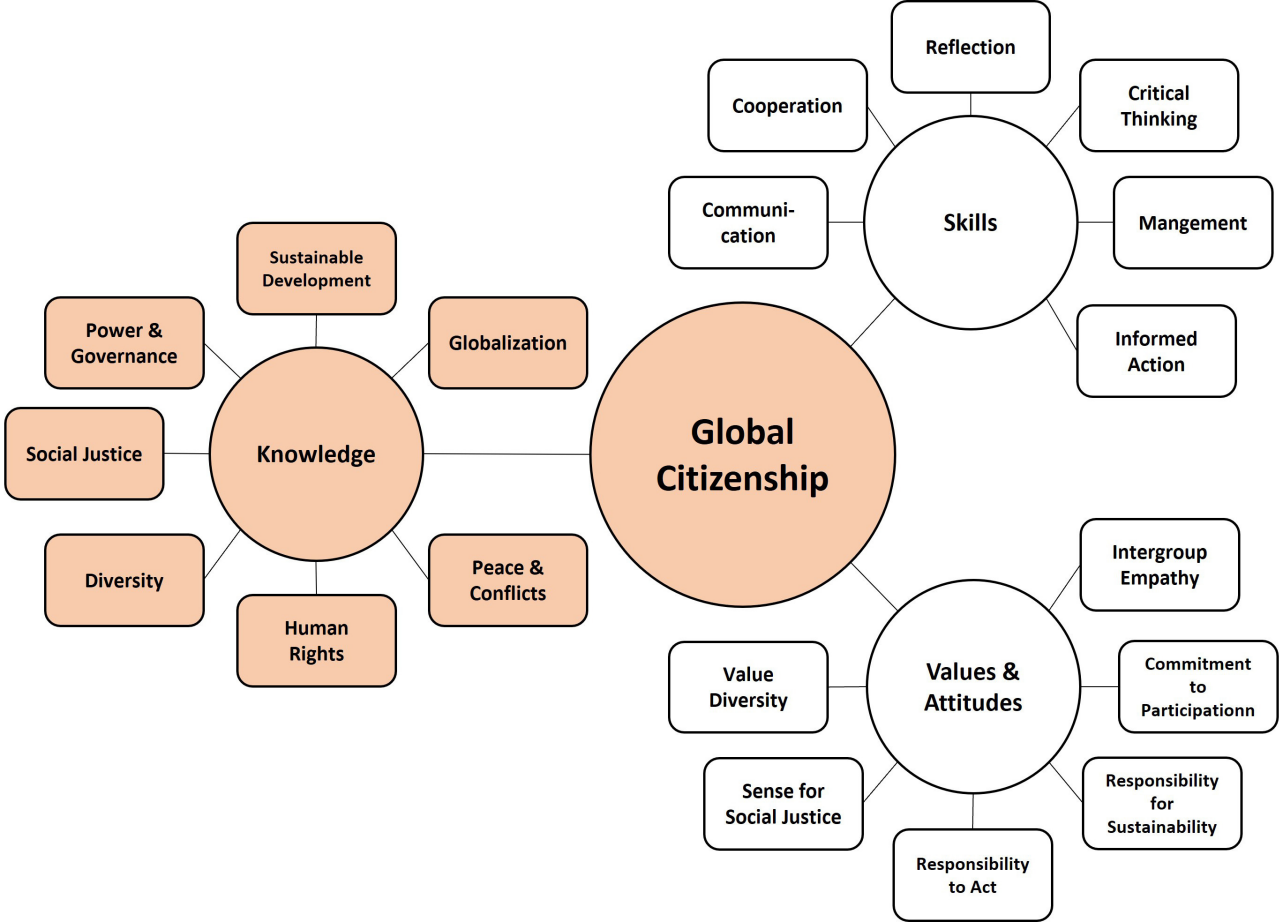
Visualization of global citizenship. Content of the three domains is derived from Oxfam (2) and Fabrigar et al. (17). Only knowledge is colored here because we refer to knowledge in this paper.

*Global Citizenship* (GC), also known as *Cosmopolitanism*, goes back to ancient Greece and targets the idea of being a citizen of the world rather than of just one nation. While no universally accepted definition exists, scholars and organizations agree that global citizens should incorporate specific values, skills, and knowledge about the functioning of the world to change the world into a better place to live (2, 18, 33). Unsurprisingly, the UN adopted GC as an “umbrella term for social, political, environmental, and economic actions of globally minded individuals and communities on a worldwide scale” (34), and various organizations developed GC education (GCE) frameworks. While scholars largely agree on a broad definition of GC, they differ on the characteristics that people should acquire to become global citizens [see Oxley and Morris (35) for an overview]. It goes beyond the scope of this contribution to disentangle different GCE frameworks, but on a certain level they entail jingle issues. Typically, skills, values, and knowledge include respect for diversity and other cultures (2, 23, 36), respect for the rights of other people, communication skills, intercultural skills (2, 36, 37), commitment to social justice (2, 19), civic action and political participation (2, 19, 36), as well as global awareness and knowledge about the world (2, 19, 37). Oxfam (2) introduced the most comprehensive GCE framework we know, differentiating seven different skills, seven different values, and six areas of knowledge (see Figure 1) students should acquire. As this paper focuses on knowledge, only the latter will be considered here.

GC knowledge is not equivalent to SD knowledge and, thus, does not adopt the three dimensions of SD (environmental, economic, and social issues). The six knowledge domains mentioned in GCE frameworks are i) s*ocial justice and equity*, ii) *identity and diversity*, iii) *globalization and interdependence*, iv) *sustainable development, peace and conflict*, v) *human rights*, and vi) *power and governance*. Most of these domains target knowledge about human rights, governance, and social systems, and only one domain targets sustainable development. Subdomains of the knowledge domains are not specified and only examples are given of what knowledge should be required in different age groups, not fulfilling exhaustiveness and disjunction criteria.

Concluding, GC frameworks highlight the importance of knowledge about the world [e.g., (2, 4)]. GCE frameworks do not explicitly mention global issues but list broad, somewhat vague knowledge domains, leaving room for interpretation. Instead of focusing on global issues, GC focuses on educating humans and fostering their knowledge to empower them to engage for a better world. This implies knowledge about the bigger picture and detailed knowledge about specific aspects and issues.

#### 2.1.2 Sustainable development/sustainability

Initially introduced in “Spaceship Earth” as balance between economic growth and resource availability in closed systems (38), our contemporary view of SD is rooted in economics. SD gained prominence when the UN adopted SD in 1987 and defined it as “development that meets the needs of the present without compromising the ability of future generations to meet their own needs.” (39). Since then, various disciplines have, redefined, and operationalized SD. Nowadays, most scholars agree on the multidimensional conceptualization linking *economic, environmental*, and *societal* factors (40–42). Authors use terms like “dimension,” “pillar,” “aspect,” “perspectives,” and “components” to describe these factors, all of which are mostly used synonymously throughout the literature (43).

**Figure 2:**
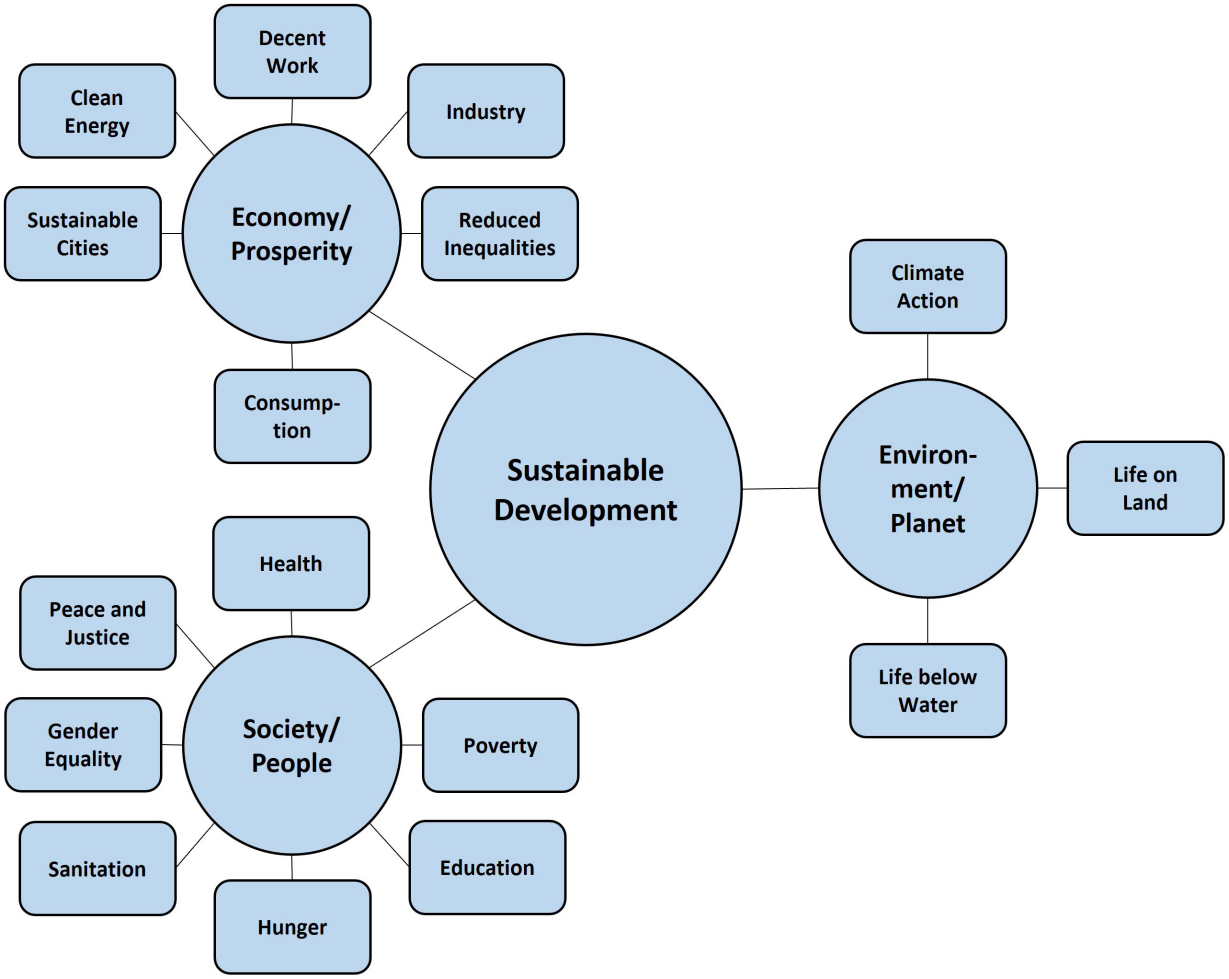
Visualization of three dimensions of sustainable development and the SDGs. The subdomains assigned to the three dimensions of SD correspond to the SDGs excluding goal 17 as an enabling goal.

SD gained further popularity when the UN introduced the SDGs as successor to the *Millennium Development Goals*. The SDGs represent the most extensive compilation of global remedies (5). The SDGs outline global issues in targets to be reached by 2030 in a seemingly hierarchical structure with 17 overarching policy goals, 196 targets (Figure 2), and 231 unique indicators to monitor the progress in SD (44, 45). While the goals align with the three pillars of SD, the UN proposed another allocation toward the five Ps: i) People: focusing on humans and human development (SDGs 1, 2, 3, 4, 5, 6), ii) Prosperity: focusing on economic, social, and technological progress (SDGs 7, 8, 9, 10), iii) Planet: focusing on the environment, climate change, and nature conservation (SDGs 11, 12, 13, 14, 15), iv) Peace: focusing on building and maintaining peace (SDG 16), and v) Partnership: focusing on building and strengthening international partnerships (5).

The SDGs are highly interrelated. Indeed, some targets and indicators are allocated to more than one goal. Other aspects are allocated to multiple goals, thereby hampering unequivocal interpretation. For instance, “equality” is targeted in terms of gender equality in SDG 5 and in terms of international equality in SDG 10. Additionally, other goals, e.g., SDG 1, 2, 4, 6, 8, and 11 address equality, which aggravated distinguishing between goals. These substantial overlaps are not surprising, as the strong interweaving of the three dimensions of SD constitutes this approach and has been subject to research [e.g., (46–48)]. These highly interconnected goals are a framework that is predominantly conceptualized for policymakers. The interconnectedness is useful as tackling one issue will automatically partially address other issues (48). However, given the partially high level of redundancy between the goals, the SDGs are not suitable as a taxonomy for knowledge about the world. Also, in other aspects, this approach deviates from stratification to provide categories of knowledge about the world. For instance, goal 17 only targets policy strategies to strengthen international partnerships. This category falls short of the breadth of the first *p* (People), which summarizes goals with a much broader scope. Thus, we take the SDGs as an outstanding compilation of major challenges for humanity. However, due to the high level of redundancy within the SDGs, we do not think the policy taxonomy's internal structure is appropriate for developing a knowledge taxonomy.

#### 2.1.3 Planetary health

**Figure 3:**
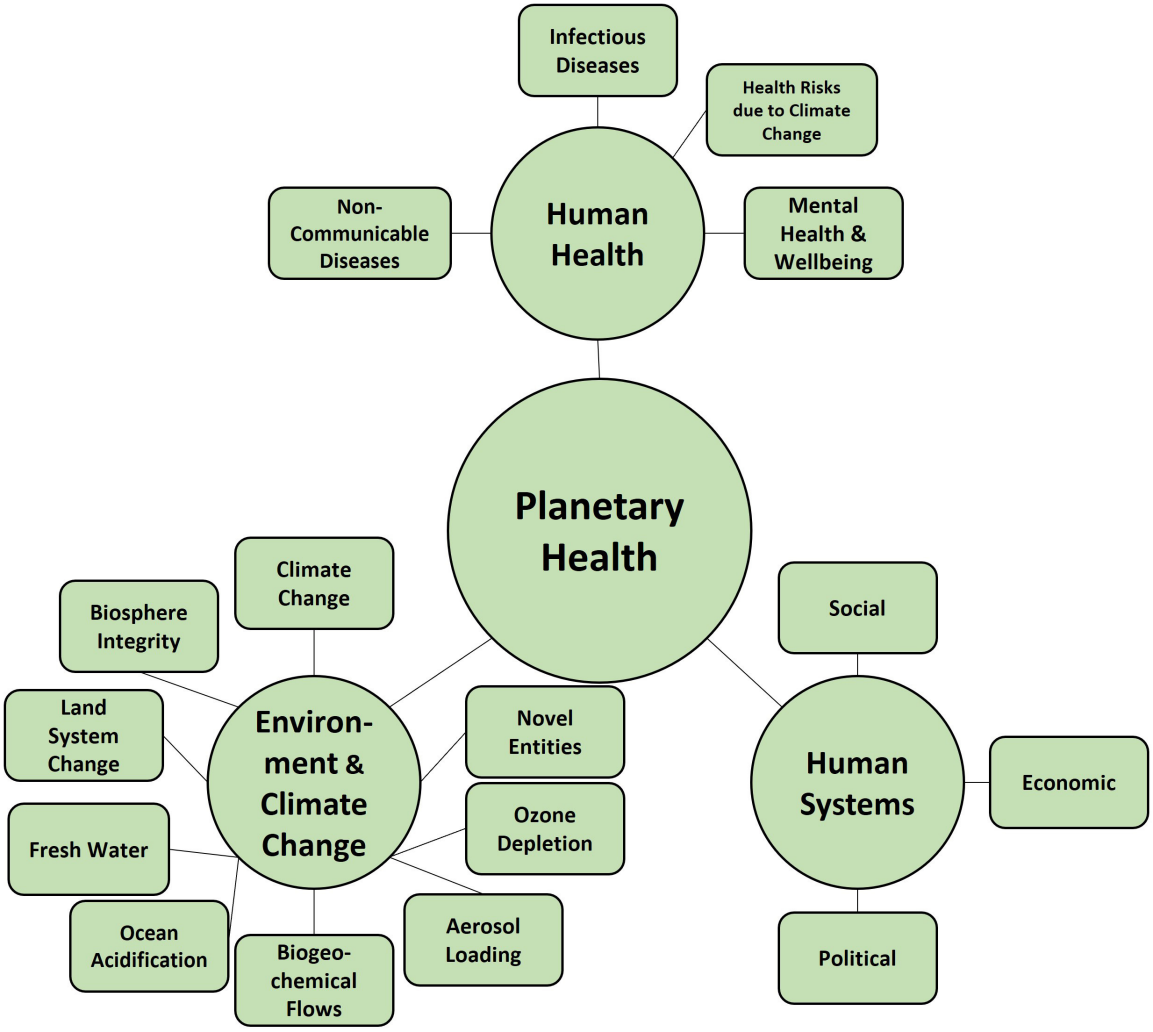
Visualization of aspects of planetary health. Content is derived from Whitmee et al. (29) and Guzmán et al. (6).

*Planetary Health* (PH) is directly linked to SD and is defined as “the health of human civilization and the state of the natural systems on which it depends” (29). PH emphasizes the interactions between human health, the global environment, and human systems (economic, political, and social) and that a healthy environment determines human life—in terms of health and social life. Additionally, it goes beyond the SDGs and stresses that achieving PH is a prerequisite to achieving SD (29). This holistic approach focuses on the interrelations of the natural environment, human health, and human systems. PH integrates different approaches and acknowledges the SDGs, the planetary boundaries (49, 50), and other approaches that focus on human health in interaction with environmental change. The absolute focus of PH lies in the inextricable link between human health and the health of planetary systems on which human civilization depends. With this focus, it is not surprising that aspects of human health and the health of the planet outweigh human systems, industry, and politics. At the level of the health of the planet, PH mentions the nine planetary boundaries: *Climate change, Ocean acidification, novel entities, ozone depletion, aerosol loading, biogeochemical flows, freshwater use, land-system change*, and *biosphere integrity* (49, 50) (Figure 3). PH further highlights points of interaction between human health (concerning infectious diseases, mental health and wellbeing, and non-communicable diseases) and acknowledges aspects of physical activity and nutrition. Finally, it also links to the SDGs, emphasizing that while the SDGs comprise an extensive compilation of issues, they lack overarching connections so that a coherent picture does not emerge. Linking to GC, a PH education framework was developed as a special kind of GCE (6), seeing the need to educate people to change developments in planetary health. This framework emphasizes the interconnections between the natural environment and human health, highlighting the impact of human action on both ecological systems and health outcomes and the importance of seeing the complete system rather than just parts of it. It further stresses respect for human and natural rights and the development of skills, knowledge, and values that enable active participation and engagement of knowledge to be acquired but highlight learning objectives. PH, SDG or GC learning objectives [e.g., (51)] bear some relevance for the taxonomy we discuss. Indeed, it would be helpful if instructional efforts would show some alignment with the taxonomy. Future research should consider concepts of curricular validity (52) when specifying interventions. We see considerable potential in classifying instructional efforts into an overarching taxonomy because this can highlight how exhaustive and profound a specific intervention covered PHK in its broadest sense.

#### 2.1.4 GC, SD, and PH concerning the criteria for taxonomies

Evaluating the three approaches regarding the criteria, taxonomies should meet, we find differences in terms of exhaustiveness. The SDGs are the most exhaustive, presenting a large collection of global issues in environmental, economic and social factors. We find none of the three approaches to be disjunctive in terms of each element only being allocated to one factor or goal. All three approaches have a different focus and different underlying goals. In GC, the focus lies on characteristics of human beings, while the SDGs define specific political goals and targets among environmental, economic, and societal factors. PH goes one step further, focusing on the interactions and evolvement of the environment, human health, and civilization. All three approaches are useful, as they present important societal issues. Additionally, each approach presents another view on the interaction of environmental, economic, and societal issues. In terms of parsimoniousness, the SDGs is the least parsimonious with 17 goals. GC is more parsimonious with six knowledge domains, but on the other hand, not as exhaustive as the SDGs. Last, it is difficult to evaluate the replicability of the approaches. Given the lack of disjunction, it might be difficult to replicate the allocation of the three approaches. Taken together, each approach focuses on the interaction of the environment and human systems from another perspective, but no single framework meets the criteria for taxonomies completely.

### 2.2 Mapping SD, GC, and PH

Based on this review, it becomes obvious that all three approaches consider dimensions of the environment and human civilization (in matters of economy, politics, social life, and human health) and interactions of environment, and human life to different extents. Although the focus and the degree of specification of the mentioned aspects differ across the approaches, all three deal with more or less the same topics. It is difficult to quantify the extent to which each approach addresses the different aspects, which is also contingent upon the construct's operationalization. Consequently, it is also difficult to quantify the degree of overlap between the three approaches. On the level of overarching dimensions, SD, PH, and GC target the same things, indicating interdisciplinary jangle fallacies.

Aiming to make the overlaps visible on a more granular level, we mapped elements with the same focus across the elements using the SDG goals as anchors. As expected, we found an immense level of redundancy. Each approach focuses on elements within the dimensions environment, economy, and human systems. At the level of the SDG goals, we found elements of nearly all goals in all three approaches. Only the goals 3 (Good Health and Wellbeing) and 7 (Affordable and Clean Energy) were not integrated in GC, whereas goal 5 (Gender Equality) was not integrated in PH. All other goals were part of all three approaches (Table 1).

**Table 1:** Comparison of sustainable development, global citizenship, and planetary health.

**SDG goal**	**Sustainable development**	**Global citizenship**	**Planetary health**
Focus of the construct	Focus on achieving a balance between economic growth, social inclusion, and the environment. The SDGs address global issues from a political perspective.	Focus on rights and responsibilities beyond the national citizenship and on the acquirement of knowledge, skills and attitudes.	Focus on the interactions of the environment, human health, and human civilization and on changed requirements, especially due to the climate change.
1. No poverty	End poverty in all its forms everywhere	Causes and effects of poverty, inequality and exclusion; measuring poverty; methods to reduce poverty [(2), p. 16]	People in poverty; Poverty and risk from environmental change; Impact of poverty on health, wellbeing, risk from climate change, and undernutrition [(29), p. 1986–7]
2. Zero hunger	End hunger, achieve food security and improved nutrition, and promote sustainable agriculture	Nutritional needs, global food distribution and markets, factors affecting nutrition, development of food markets [(2), p. 10–2]	Over- and Undernutrition, impact of climate change on nutrition and food security; impact from agriculture on soil erosion; impact from climate change on agriculture [(29), p. 1978].
3. Good health and wellbeing	Ensure healthy lives and promote wellbeing for all at all ages	N.a.	Human health impacts of environmental changes (infectious diseases, non-communicable diseases, mental health and wellbeing), and adaptations of health systems adaptation (29)
4. Quality education	Ensure inclusive and equitable quality education and promote lifelong learning opportunities for all	GC and GCE focus on educating people of all ages to become global citizens. The complete concept is focused on education and the acquisition of required knowledge, skills, and values.	Role of education in promoting health, interconnection of education and health [(29), p. 1996, 2015]
5. Gender equality	Achieve gender equality and empower all women and girls	Gender equality in different aspects of social life, global gender disparities, gender stereotypes [(2), p. 10, 13, 16]	N.a.
6. Clean water and sanitation	Ensure availability and sustainable management of water and sanitation for all	Need of water, water conservation [(2), p. 10]	Interactions between environment, sanitation, freshwater availability, health, and food security [(29), p. 1974, 1984]
7. Affordable and clean energy	Ensure access to affordable, reliable, sustainable, and modern energy for all	N.a.	Interactions between clean energy, air pollution and human health [(29), p. 2004]
8. Decent work and economic growth	Promote sustained, inclusive, and sustainable economic growth, full and productive employment, and decent work for all	Knowledge of different rights, different economic systems, economic factors for nutrition, inequalities, and global development [(2), p. 13, 16–7]	Interactions between economic growth, poverty, environment (especially water scarcity), and health [(29), p. 1980]
9. Industry, innovation, and infrastructure	Build resilient infrastructure, promote inclusive and sustainable industrialization, and foster innovation	Impact of technology on development and quality of life, sustainable industrial practices [(2), p. 12]	Environmental and health impacts of industry and infrastructure; Need for sustainable industry and infrastructure [(29), p. 1980–1]
10. Reduced inequality	Reduce inequality within and among countries	Global inequalities, causes and consequences of inequalities; measuring inequality; inequality in relation to exclusion, discrimination, politics, power, and social systems; reduction of inequalities, social justice; diversity of people; cultures and societies; identity [(2), p. 16–7]	Interactions of Inequality with economic, environmental, and social factors [(29), p. 2011]
11. Sustainable cities and communities	Make cities and human settlements inclusive, safe, resilient, and sustainable	Globalization, differences and similarities of different places in the world, interdependencies between global and local issues [(2), p. 16]	Complex interactions between urbanization, health, environment [(29), p. 1985]
12. Responsible consumption and production	Ensure sustainable consumption and production patterns	Different lifestyles and consumerism; global impact of lifestyles; changing lifestyles and consumerism; environmentally-responsible living [(2), p. 18]	Impact of modern production on the environment and human health; Sustainable resource use [(29), p. 1981]
13. Climate action	Take urgent action to combat climate change and its impacts	Climate change awareness, global and local causes and implications of climate change [(2), p. 16]	Climate change in all possible aspects (e.g., planetary boundaries) and effects on human health and civilization (29)
14. Life below water	Conserve and sustainably use the oceans, seas, and marine resources for sustainable development	Biodiversity; Taking care of the environment; basic needs of living organisms; water conservation; Sustainable Development Goals, environmental impact of technological discoveries and developments; dilemma of development and sustainability [(2), p. 16]	Interaction of human activities, oceans' health, marine ecosystems and species, and human health and nutrition (29)
15. Life on land	Protect, restore, and promote sustainable use of terrestrial ecosystems, sustainably manage forests, combat desertification, and halt and reverse land degradation and halt biodiversity loss		Complex interaction between human activities, environmental change, changes in land use and ecosystems and human health in different aspects (29)
16. Peace, justice, and strong institutions	Promote peaceful and inclusive societies for sustainable development, provide access to justice for all, and build effective, accountable, and inclusive institutions at all levels	Human rights (HR), causes and implication of HR violations; justice and peace; peace making and peace keeping; global and national governance [(2), p. 15–6]	N.a.

Goal 17 was excluded as it is an enabling goal and does not target global issues. N.a. means that the content is not addressed in the respective construct.

### 2.3 Discussion

Summarizing, all three approaches have somewhat different origins and purposes but tackle essentially the same topics that constitute major challenges of humanity. They differ in term of exhaustiveness with the SDGs presenting the largest collection of global issues and PH going beyond the SDGs by focusing on the interactions of single elements. None of these approaches can be readily turned into a PHK taxonomy that could serve as a blueprint for developing and evaluating measures of knowledge. Besides, the high level of redundancy across approaches indicates the existence of jingle issues. This hampers interdisciplinary collaboration and communication. Resolving such issues could greatly facilitate communication and collaboration about the ongoing problems. As a step in this direction, we decided to combine, adjust, and realign the above taxonomies into a taxonomy that presumably adheres to the criteria for a sound taxonomy described above (exhaustiveness, disjunction, usefulness, parsimoniousness, and replicability).

## 3 Planetary health knowledge taxonomy

### 3.1 Development procedure

The taxonomy development is based on the SDGs (excluding goal 17 as an enabling goal) as they constitute the largest and earliest collection of global issues. Starting with the SDGs, we extracted elements from the targets, divided multi-aspect targets into distinct elements, and eliminated redundant elements (i.e., elements appearing multiple times). These elements were grouped into small thematic clusters to form facets, which were grouped into larger clusters to define subdomains, which were finally grouped into larger clusters to define domains. To ensure replicability and disjunction within the taxonomy, we followed a systematic process. We made key decisions, such as how to handle gender equality. The SDGs treat gender equality in a separate domain and subordinate in other domains like Education. This caused duplication of content, so we listed gender equality only as subordinate where applicable. We applied the same approach for other groups, like age or ethnicity.

Afterward, two independent raters assigned elements to these categories. We analyzed interrater reliability and resolved discrepancies through discussion that led to adjustments such as refining definitions or modifying subdomains. Elements with inconsistent prior assignments were reassigned and discussed, again. For example, the availability of safe public green spaces in cities was allocated to P3 *Environment* by one rater, while the other rater allocated it to P6 *Standard of Living*. According to the initial definitions of the respective domains before interrater study, both allocations would have been correct. While P3 focuses on the environment, covering among others, green spaces in the context of renaturalization and biodiversity, P6 focuses on people, covering the existence and use of safe and pleasant retreats, among other things. After discussions, we concluded that “safe public green spaces” belong to P6 as its focus is predominantly on people's safety and wellbeing. Thus, we tightened up the definitions of the respective domains and subdomains, specifying the focus more clearly. We repeated this process until achieving 100% interrater reliability (see Supplementary material). The subdomains were further enriched with elements extracted from PH and GC. Finally, we added essential content and elements inspired by initiatives such as the Dollar Street project (53).

### 3.2 PHK taxonomy

**Figure 4:**
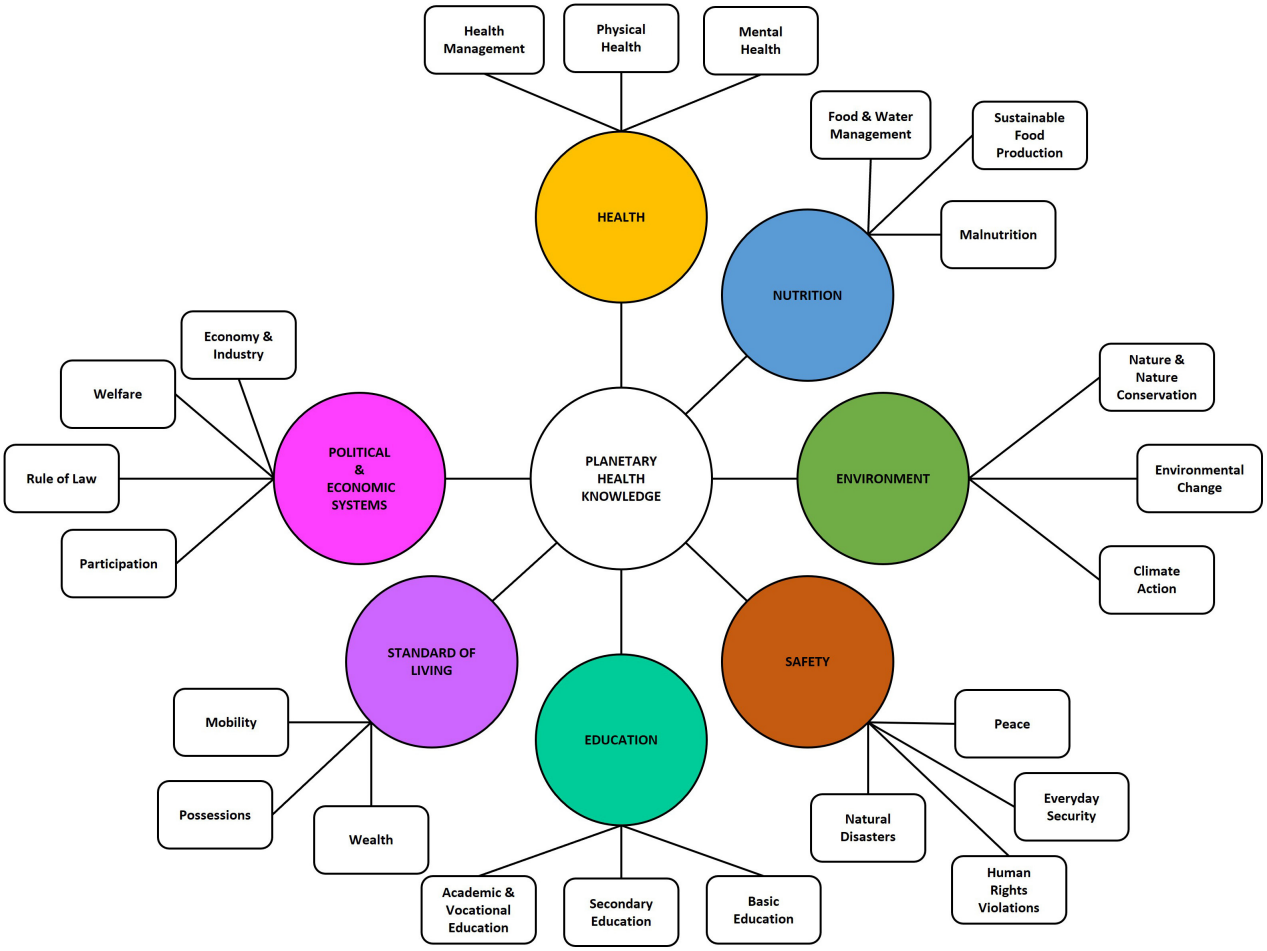
Visualization of the planetary health knowledge taxonomy on subdomain level.

The resulting taxonomy comprises seven domains: i) *Health*, ii) *Nutrition*, iii) *Environment*, iv) *Safety*, v) *Education*, vi) *Standard of Living*, and vii) *Political and Economic systems* with three or four subdomains each (Figure 4). These domains all possess broad scope and align closely with conceptualizations in PHK, SDG, and GC, while maintaining thematic separation to the greatest extent possible. The following section provides a detailed description of the domains, sub-domains and associated content. A brief overview of their scope is given in Table 2.

**Table 2:** Planetary health knowledge: domains, subdomains, facets, and content.

**Domain**	**Subdomain**	**Facet**	**Content (examples)**
P1 Health	A Health management	Risk management	The global and local handling of epidemics and pandemics
		Health coverage	The distribution of trained healthcare professionals across the globe
		Prevention	The role and availability of sanitary facilities and effective prevention methods
		Treatment	The role and availability of effective drugs to tackle diseases, and distribution of antimicrobial resistances
	B Physical health	Infectious diseases	The prevalence, treatment, transmission, and spread of major infectious diseases as HIV, malaria, and tuberculosis
		Non-communicable diseases	The spread and prevalence of non-communicable diseases such as cancer, cardiovascular diseases, diabetes and chronic respiratory diseases as well as risk factors and consequences
		Birth risks	Risk factors of child and maternal mortality in connection with births and possible solutions
	C Mental health	Substance abuse	Where substance abuse is particularly prevalent, groups at risk of substance abuse, total prevalence, consequences for impaired people and relatives, discrimination associated with substance abuse
		Affective disorders	Prevalence of affective disorders, in which places affective disorders are most prevalent, risk factors for the manifestation, associated discrimination, consequences for impaired people and relatives
		PTSD and wellbeing	Where and to which extent is PTSD the most prevalent, causes and consequences of PTS, causes of high wellbeing, relations of wellbeing to other domains and facets
P2 Nutrition	A Food and water management	Food and water availability	Distribution of food and water around the world, causes for unavailability and inaccessibility of sufficient food and water, risk factors for not having sufficient food and water
		Food and water affordability	Reasons for non-affordability of food and water, the link between hunger and poverty, areas with highest share of people who cannot afford sufficient food, correlations with political and economic systems beyond this
		Food and water quality	Criteria of quality and uncontaminated food, areas with high and low-quality food, causes that lead to contamination, types of storage that makes food more durable, the composition of healthy meals
	B Sustainable food production	Agriculture	The amount of land needed for different types of agriculture, CO_2_ emissions of different farming methods, organic farming with its consequences
		Fisheries	The sustainability of different fishing methods, consequences of industrial fishing for life (bycatch) and habitat, possible ways to make fishing mor sustainable and environmentally sound
		Livestock	Environmental impact of livestock, areas where livestock farming is particularly prevalent, the amount of water/food/agricultural land needed for meat “production,” consequences of organic livestock farming
	C Malnutrition	Under- and malnutrition	Prevalence, causes, and consequences of malnutrition and unbalanced diets that lead to nutrient deficiencies
		Obesity	Prevalence, causes, and consequences of overweight and obesity, stigmatization due to obesity, and consequences for general wellbeing and physical and mental health
P3 Environment	A Nature and nature conservation	Biodiversity	Current status of biodiversity in the animal and plant world, causes and consequences of the decline and the role of biodiversity for different ecosystems
		Protected areas	What kind of land and water areas are protected and to what extent, what happens in protected areas and what is to be achieved
		Ecosystems	The function of ecosystems for nature, the current state of ecosystems, and the distribution of disturbed ecosystems
	B Environmental change	Pollution	Water, air and soil pollution, and main causes and effects of pollution
		Waste handling	Waste production in different countries, handing of waste and the significance of waste handling for individual countries and the environment
		Climate change	How and where climate change manifests in which extent and its consequences and causes
		Extreme weather events	The increasing occurrence of extreme weather events such as droughts, floods, and heatwaves due to climate change as well as the impact for people and the environment
	C Climate action	Climate target plans	Political climate goals and the effects they should have
		Renewable energies	The spread of renewable energies and their impact on the environment compared to conventional energy sources
		Resource efficiency	The efficacy and functionality of energy sources and ways to use a minimum of natural resources
		Climate goals	Political measures to counteract the climate change, effective measures that can be taken by individuals to contribute to climate protection
P4 Safety	A Peace	War	Which regions are affected by war and the impact this has on lifestyle, health, and prospect for the future
		Organized crime	Regions affected by organized crime underlying groups and structures, the impact on the population risk factor of being involved into organized crime
		Terrorism	Differentiation of organized crime and terrorism, regions that are most affected by terrorism and organized crime, impact on citizens, strategies for combatting terrorism
	B Everyday security	Occupational security	Safety measures at work, legal safeguards, occurrence of serious work-related accidents, consequences of an unsafe working environment
		Cyber security	Different types of cyber security, extent and quality of exposure in private and work contexts, protection measures against cybercrime
		Violence	The occurrence of domestic or sexual violence in different countries, violence against children, effects on victims and perpetrators, measures to protect against violence
	C Human rights violations	Non-Discrimination and Equality	Extent and distribution of discrimination based on gender, ethnicity, religion etc. in fields as education, property rights, persecution or expulsion, general knowledge toward the declaration of human rights
		Freedom violations	Extent, distribution and significance of restrictions on freedom of expression, freedom from slavery and human trafficking and measures to combat freedom violations
		Other human rights violations	Extent and distribution of other human rights violations, impact for affected people, consequences for victims and perpetrators and possible solutions
	D Natural disasters	Resilience building	Different kinds of early warning systems, importance of early warning, existence of warning systems and correlation with country's prosperity, measures such as earthquake-resistant construction
		People exposed	Prevalence of affectedness (mortality, injuries, loss of housing) in various natural disasters and the significance for affected people
		Damage caused	Amount of economic loss, damaged or destroyed infrastructure, humanitarian situation and mitigation measures
P5 Education	A Basic education	Literacy and numeracy	The prevalence of said skills, the importance of these basic skills for personal development, correlations with social status
		Primary education	Availability and attendance of elementary schools, equality in primary education
		Affordability and quality	Cost of education (paid by the state and the individual), state and private school systems, quality of education, structural problems such as teacher absenteeism and training
	B Secondary education	Attendance and availability	Frequency of secondary degrees, gender equality in attendance and degrees, importance of secondary degrees for social mobility
		Affordability and quality	Costs covered by parents, quality differences between countries with reasons and causes, structural problems in secondary education
	C Academic and vocational education	Academic (tertiary) education	Prevalence of university degrees, equality within and between countries, career prospects, importance for social status and personal development, chances to reach tertiary education
		Vocational education	Different systems of vocational training, frequency of degrees and certificates, importance for personal development and prosperity
P6 Standard of living	A Wealth	Income	Average income in purchasing power, relations with health and education
		(Un-)employment	Extent of unemployment, correlations with happiness, development of criminality
		Social protection	Extent to which people have and make use of social security, significance for wellbeing, standard of living, and other factors
	B Possessions	Sanitation	Presence of sanitary facilities in the private space, and in public spheres, different kinds of sanitation facilities
		Home possessions	Possessions, people have in different places, possessions deemed to be status symbols, meaning of possessions in different countries and societies
		Housing situation	Different types of housing, space per person, energy consumption, materials from which the home is built, sustainable housing
	C Mobility	Availability	Accessibility of all-year roads and public transport systems the importance of connectivity for social status, job opportunities, links to environmental factor
		Affordability	Extent to which private and public mobility can be afforded, causes and consequences of non-affordability
		Traffic rules and security	Existence of traffic rules, prevalence of accidents and deaths by accidents, relation of accidents and traffic rules, adherence to traffic rules
P7 Political and economic systems	A Participation	Elections and voting rights	Free election, equality in voting rights, impact on and from human rights, relation to wealth and prosperity
		Leadership positions	Equality in leadership positions, opportunities for career advancement
		Inclusion in governments	Composition of governments, formation of governments and opportunities to take leadership roles in politics
	B Rule of law	Judicial systems	Principles of jurisdiction, functioning of the judiciary, corruption in the judicial system
		Form of government	Different kinds of governments and states, importance for citizens and international relations
		Laws to protect human beings	Laws introduced in order to protect people, which countries have laws adopted and to which extent, what kind of laws have been introduced in various areas, e.g., to stop corruption and to enhance labor security
	C Welfare	Social security	Existence and financing of insurance and social benefits, relevance for a perception of social security
		Subsidized social services	Which benefits are state-subsidized, financing of these benefits, relevance for prosperity at state and individual level
	D Economy and industry	Economy	Economic developments, different kinds of economic systems, international economic relations
		State-level prosperity	Wealth of different states, interaction with economic and state systems, inequality of wealth in society
		Industry and production	Developments in industry and production, quantity and diversity of industry, relations of economic standards and industry, research on technology development

#### 3.2.1 Health

The domain of *Health* focuses on physical and mental health, as well as global challenges in healthcare and health management. It comprises three subdomains. *Health Management* refers to challenges in global healthcare with four facets: *Risk Management* (e.g., the global and local handling of epidemics and pandemics), *Health Coverage* (e.g., global distribution of trained healthcare professionals), *Prevention* (e.g., role and availability of sanitary facilities), and *Treatment* (e.g., access to effective drugs to tackle diseases, and distribution of antimicrobial resistances). *Physical Health* focuses on major physical diseases with three facets: *Infectious Diseases* (e.g., the prevalence, treatment, transmission, and spread of infectious diseases such as HIV, malaria, and tuberculosis), *Non-Communicable Diseases* (e.g., spread and prevalence of non-communicable diseases such as cancer, cardiovascular diseases, diabetes, and chronic respiratory diseases including risk factors and consequences), and *Birth Risks* (e.g., child and maternal mortality including risk factors and possible solutions). *Mental Health* addresses the status quo of global mental health with four facets: *Substance Abuse, Affective Disorders, Post-Traumatic Stress Disorder* (PTSD), and *Wellbeing*, all referring to the distribution of mental illnesses including associated risk factors, impairment, and discrimination.

#### 3.2.2 Nutrition

The domain of *Nutrition* focuses on the global distribution and production of food and water, malnutrition, and health-related aspects of nutrition It consists of three subdomains. *Food Management* targets the availability, affordability, and quality of food and water with associated causes and possible solutions in three facets: *Food Availability* (e.g., distribution and causes of food shortages), *Food Affordability* (e.g., Economic access to food and water with associated risk factors), and *Food Quality* (e.g., Nutritional value, food storage and durability, balanced diets). *Sustainable Food Production* examines agriculture, fisheries, and livestock in terms of environmental impact and sustainability, with three facets: *Agriculture* (e.g., Land use, and CO_2_ emissions of different agricultural methods), *Fisheries* (e.g., Sustainability of different fishing practices including destruction of life (bycatch) and habitat), and *Livestock* (e.g., Environmental impact and resource requirements). *Malnutrition* focuses on health-related issues related to nutrition, implying a linkage to *Health*. *Under and Malnutrition* (e.g., prevalence, causes, consequences, and different forms of nutrient deficiencies) and *Obesity* (e.g., prevalence, causes, and consequences of overweight and obesity and possible countermeasures).

#### 3.2.3 Environment

The domain of *Environment* addresses ecosystems, biodiversity, climate change, and climate action, organized into three subdomains. *Nature and Nature Conservation* focuses on the current state and changes of various ecosystems, biodiversity, and nature restoration in three facets: *Biodiversity* (e.g., Current status, decline causes and consequences of biodiversity in flora and fauna, and the role of ecosystems), *Protected Areas* (e.g., Extent, management, and goals of protected regions and areas), and *Ecosystems* (e.g., Function, state and disruption of ecosystems). *Environmental Change* examines environmental pollution and climate change impacts in four facets: *Pollution* (e.g., Water, air, and soil pollution, including causes and effects), *Waste Handling* (e.g., Global waste production and management and its impacts), *Climate Change* (e.g., Manifestations, causes, and consequences of climate change), and *Extreme Weather Events* [e.g., (increasing) Frequency and impacts of weather extremes]. *Climate Action* addresses political and individual measures to mitigate climate change in four facets: *Climate Target Plans* (e.g., Political climate goals and expected effects), *Renewable Energies* (e.g., Spread of renewable energies and environmental impact compared to conventional energy sources), *Resource Efficiency* (e.g., Minimizing natural resource usage including efficacy and functionality of energy sources), and *Climate Goals* (e.g., effective policy and citizen measures to contribute to climate protection).

#### 3.2.4 Safety

The domain of *Safety* focuses on creating secure and peaceful environments, with four subdomains: *Peace* targets large-scale and impactful safety issues in three facets, differentiating wars as territorial conflicts, organized crime with mainly economic motivation, and terrorism as ideological conflicts: *Wars* (e.g., affected regions and impact on lifestyle, health, and future prospects), *Organized Crime* (e.g., affected regions, underlying groups and structures, the impact on the population risk factor of being involved in organized crime), and *Terrorism* (e.g., Regional distribution, impact, possible counter-strategies, differentiation from organized crime). *Everyday security* covers daily safety issues in three facets: *Occupational Security* (e.g., prevalence of work-related severe accidents and impact, workplace safety, legal safeguards, prevalence), *Cyber Security* (e.g., types, extent and quality of exposure, and protection measures), and *Violence* (e.g., Prevalence, effects, and protective measures against domestic violence). *Human Rights Violations* focuses on human rights, human rights violations and human rights protections in three facets: *Non-Discrimination and Equality* (e.g., prevalence, distribution, and impact of discrimination on various reasons and in various contexts; property rights, persecution or expulsion; general knowledge toward the declaration of human rights), *Freedom Violations* (e.g., extent, distribution, and significance of different freedom and countermeasures), *Other Human Rights Violations* (e.g., extent, consequences, and solutions of other human rights violations). *Natural Disasters* examines the impact of different natural disasters on human beings in three facets: *Resilience Building* (e.g., Availability of warning systems, preparedness, and solutions), *People Exposed* (e.g., prevalence of affectedness (mortality, injuries, loss of housing) and impact on affected populations), and *Damage Caused* (e.g., economic losses, humanitarian consequences, other impact, and mitigation measures).

#### 3.2.5 Education

The domain of *Education* focuses on global access, quality, availability of education, educational opportunities, and the role of education in social mobility issues in three subdomains. *Basic Education* addresses literacy and numeracy, and affordability and availability of primary education in three facets: *Literacy and Numeracy* (e.g., prevalence, correlations with social status, impact on personal development), *Primary Education* (e.g., access, attendance, and equality of elementary schools and early childhood education), and *Affordability and Quality of education* (e.g., Educational costs (covered by state or family); structural issues, different school systems, quality of education, teacher training). *Secondary Education* examines attendance, affordability, and quality of secondary education in two facets: *Attendance and Availability* (e.g., distribution and gender equality of attendance and degrees, importance for social mobility) and *Affordability and Quality* (e.g., educational costs (covered by state or family); structural disparities, quality differences between countries including causes and consequences, structural problems in secondary education). *Academic and Vocational Education* focuses on academic and non-academic vocational education in two facets: *Academic (Tertiary) Education* (e.g., prevalence of university degrees, equality within and between countries, impact for social mobility and personal development), and *Vocational Education* (e.g., different vocational education systems, distribution of degrees and certificates impact for personal development).

#### 3.2.6 Standard of living

The domain *Standard of Living* focuses on individual prosperity worldwide with three subdomains. *Wealth* targets individual financial aspects in three facets: *Income* (e.g., Distribution of average purchasing power, links to health and education), *Unemployment* (e.g., Extent and impact, e.g., on happiness and crime), and *Social Protection* (e.g., Access to social security, impact on e.g., wellbeing and social status). *Possessions* highlights living standards and possessions across the globe trying to catch differences in daily life in three facets: *Sanitation* (e.g., Availability of sanitary facilities at home and in public spheres; impact on spread of diseases), *Home Possessions* tackles people's standard of housing in terms of things they own and can afford and status symbols (e.g., Ownership and possessions, affordability, and status symbols), and *Housing Situation* (e.g., Housing types, space, sustainable housing, and energy use). *Mobility* addresses transportation accessibility and affordability in three facets *Availability* of transport infrastructure and public transport (e.g., Accessibility of all-year roads and public transport impact on social status and job opportunities, links to environmental factors), *Affordability* of transport infrastructure and public transport (e.g., Costs of private and public transport), and *Traffic Rules and Security* (e.g., Existence of and adherence to traffic rules, safety regulation, and traffic victims).

#### 3.2.7 Political and economic systems

The domain of *Political and Economic Systems* addresses institutional functions and infrastructures with four subdomains. *Participation* examines differences in citizen involvement, leadership, and inclusion in governments in three facets: *Elections and Voting Rights* (e.g., Free elections, equality in participation and voting rights, links to prosperity and human rights), *Leadership Positions* (e.g., Equality in leadership, opportunities for career advancement), and *Inclusion in Governments* (e.g., Government composition and political leadership opportunities). *Rule of Law* focuses on different state and judicial systems in three facets: *Judicial Systems* (e.g., Principles of jurisdiction, judiciary functioning, and corruption), *Form of Government* (e.g., Types of governments and states, impact for citizens and international relations), and *Laws to Protect Human Beings* (e.g., distribution of implementation and adoption rates of laws preventing harm, corruption, and trafficking). *Welfare* explores different welfare systems, financing, and societal impact in two facets: *Social Security* (e.g., Existence, financing, and quality of insurance and social benefits) and *Subsidized Social Services* (e.g., state-subsidized benefits, their financing and relevance for prosperity). *Economy and Industry* focuses on economic systems, developments, and problems in three facets: *Economy* (e.g., Types of economic systems and international economic relations), *State-Level Prosperity* (e.g., Wealth distribution, societal inequality, and relations to economic and state systems), and *Industry and Production* (e.g., Industrial diversity, innovation, development impact).

### 3.3 Deriving a psychometric PHK-test from the taxonomy

Developing comprehensive knowledge tests based on this taxonomy seems straightforward. We distinguish four key aspects of knowledge, all of which should be considered: i) Conceptual and factual knowledge (Fact) about the problems, ii) comprehension knowledge of causes, consequences, interrelations, and interdependencies of problems (Comp), iii) knowledge about the quantitative extent of problems (Prev), and iv) knowledge about the global distribution of these problems (Glob). In future research, we aim to establish an open, public, multilingual, and free database of knowledge items that can be used to compile knowledge tests for specific domains, inquiring about a specific form of knowledge, or tailoring a test in expected difficulty to be eligible for specific preselected samples of participants^1^.

Knowledge items can be presented in various formats, including open response (including completion) or multiple-choice (MC). Each format has distinct limitations. Open-response formats are more demanding in the evaluation, because responses need to be coded according to a coding scheme. MC items are less demanding in terms of administration since participants' task is to provide the correct response out of a number of given responses.

Additionally, items can be presented in a verbal, numerical, visual or auditory format using pictures, videos, or audio recordings (54). The impact of such format decisions the between on and data's mean or covariance structure remains uncertain, and pragmatic decisions usually favor simpler and cheaper formats (such as MC). Design features should be aligned with content. For instance, numerical stimuli suit prevalence questions, while maps seem appropriate for queries of global distribution. Beyond item attributes, alternative considerations (e.g., targeting particularly affected regions) may be pursued. Ultimately, empirical data will show if item attributes affect measurement quality.

Each item drawn from a hypothetical item universe is expected to represent PHK equally well, although different items might capture knowledge that might be deemed more or less prototypical for a field. Therefore, items can be exchanged with other items that meet the same demand as those in some inquiries. This important principle has been labeled the indifference of the indicator principle (55). Accordingly, test users can decide upon the scope of a test, more or less suited item formats, etc. Based on various considerations, we developed a broad set of multiple-choice items with verbal and numerical stimuli and we present some sample items for PHK in Table 3 (the development of this item pool and item sampling strategies will be targeted in separate contributions).

**Table 3:** Sample items for planetary health knowledge.

**Item stem**	A^*^	**B**	**C**	**D**	**Level**	**Domain**	**M (SD)**
In 2020, an average of 8 mothers died per 100,000 live births in Europe. How many died on average in Africa?	300	50	100	500	Prev	HLT	0.353 (0.170)
Antibiotic resistance means that antibiotics do not function anymore because...	Bacteria are resistant to antibiotics.	Humans are resistant to antibiotics.	Antibiotics are resistant to bacteria.	Humans are resistant to bacteria.	Fact	HLT	0.650 (0.268)
Which region was the leading producer of bananas in 2022?	South and Central America	South and Central Asia	Africa	Australia	Glob	NUT	0.699 (0.444)
Which of the following does NOT belong to the major threats to the world's oceans?	Marine tourism	Overfishing	Underwater noise	Wastewater from agriculture and industry	Comp	NUT	0.336 (0.177)
What proportion of the oceans was completely protected from human intervention at the beginning of 2023?	3%	23%	43%	63%	Prev	ENV	0.487 (0.348)
About 80% of the world's plastic waste...	Ends up in landfills or oceans.	Is being recycled.	Gets incinerated.	Ends up in forests or mountain areas.	Comp	ENV	0.708 (0.226)
Which of the following crimes is NOT a human rights crime punishable by the International Criminal Court?	Restrictions on freedom of information	Genocide	Crimes against humanity	War crimes	Comp	SAF	0.831 (0.417)
Which of the following countries is NOT one of the countries where criticism of the government is most severely suppressed and persecuted?	Bolivia	Belarus	Myanmar	Saudi Arabia	Glob	SAF	0.393 (0.291)
In 2019, around 58 million children of primary school age (5–11 years) worldwide did NOT attend school. How many girls were affected?	32 million	3 million	13 million	22 million	Prev	EDU	0.439 (0.431)
In 2018, 63 million primary school-age children were out of school. Where did more than half of them live?	Sub-Saharan Africa	Eastern Europe	South Asia	South America	Glob	EDU	0.720 (0.329)
In which places is access to safe sanitation particularly limited?	In refugee camps	In schools	In private households	In religious facilities	Fact	STD	0.747 (0.309)
The risk of poverty…	Increases with the number of children.	Is higher for men than for women.	Is higher for adults than for children.	is highest worldwide in South America.	Comp	STD	0.397 (0.207)
Which of the following countries is known for the most effective governance?	Switzerland	USA	India	Brazil	Glob	POL	0.738 (0.527)
How did the proportion of people, covered by essential healthcare, change from 2000 to 2021 worldwide?	It increased by 15%.	It increased by 35%.	It decreased by 15%.	It decreased by 35%.	Prev	POL	0.433 (0.154)

HLT, P1 Health; NUT, P2 Nutrition; ENV, P3 Environment; SAF, P4 Safety; EDU, P5 Education; STD, P6 Standard of living; POL, P7 Political and Economic Systems. Prev, Prevalence; Glob, Global Distribution; Fact, Conceptual and Fact Knowledge; Comp, Comprehension (Causes, Consequences and Interrelations). M, Mean; SD, Standard deviation. ^*^Correct.

## 4 Discussion

We propose the PHK taxonomy defining the scope of knowledge about the world's current status and global issues, including causes and consequences, to close the gap of an insufficiently defined scope. Pursuing a literature-based approach and reviewing three essential and popular approaches from different disciplines dealing with global problems of humanity, we found significant overlap and correspondence but also substantial redundancy among elements targeted by SD, PH, and GC, indicating the presence of jangle issues.

Broadly speaking, the three approaches consider the same or similar problems from different perspectives, focusing on different content and outcomes. The coexistence of different approaches that essentially address the same problems under different labels hampers interdisciplinary collaboration. The interdisciplinary nature of this field increases the need for consistent labeling of phenomena across disciplines. Divergent terminology obscures prior investigations and interventions, delaying progress and causing suboptimal use of resources such as time and money.

Comprehensive representation of environmental problems is crucial to avoid construct underrepresentation. SD, for example, is a broad and holistic approach that often faces criticism for its perceived emphasis on the economy (47). Similarly, researchers and citizens alike frequently overlook the SDG's integration of environmental, economic, and social dimensions, being only aware of one or two of the dimensions (56, 57).

### 4.1 Judging the PHK taxonomy

We have combined and reorganized elements of the three approaches discussed above to develop a comprehensive taxonomy of knowledge. This taxonomy includes knowledge from seven distinct domains. Unequivocal distinction between domains is a central characteristic of a knowledge taxonomy. However, we do not claim these domains or elements of different domains to be orthogonal—to the opposite, there are substantial interdependencies. Knowledge about elements of separate domains and about interdependencies of elements across domains can and have to be reflected in PHK knowledge tests and in potential educational programs.

The resulting taxonomy is only one way of organizing the included elements. Researchers with other perspectives would possibly come to another taxonomy. We developed the PHK taxonomy to best meet the five criteria for sound taxonomies (exhaustiveness, disjunction, usefulness, parsimoniousness, and replicability) (27, 28). Based on these criteria, we want to judge the quality of the taxonomy. Further research will be needed to evaluate the taxonomy in a data-driven manner.

#### 4.1.1 Exhaustiveness

Exhaustiveness as a criterion is probably met as we merged the most comprehensive frameworks from relevant fields. Considering additional frameworks such as One *Health* or *Buen Vivir* for taxonomy development is an option and we leave this endeavor to other researchers. We deem it unlikely, that hitherto unconsidered elements will be added to the taxonomy. Another take on exhaustiveness can be to figure in aspects of religion and culture. We consider both religion and culture as relevant context variables (58) that are not an intrinsic part of the taxonomy.

Aspects of culture and religion related to conflicts, for instance, are subordinate in the taxonomy. For example, religiously motivated conflicts are included in P4 *Safety* in the domain *Peace* in *wars* and *terrorism*, whereas religiously motivated human rights violations are included in P4 *Human Rights Violations*. There, we do not only address the existence of conflicts, *per se* but also multifactorial causes—including cultural and religious motivation.

#### 4.1.2 Disjunction

We did our best to define the domains as precisely as possible to separate them. The elements are, however, strongly intertwined, making a complete separation impossible. For example, we address inequality across all domains and subdomains wherever it makes sense instead of in a separate domain to avoid unnecessary thematic redundancy. A version of using a separate domain of inequality would result in doubling the contexts of inequality here and leaving inequality out of the other domains. This seemed awkward to us, so we decided to go with the suggested version.

#### 4.1.3 Usefulness

The proposed taxonomy is useful as it structures essential knowledge about the world, providing a foundation for applications such as developing knowledge tests and targeted interventions. These practical implications demonstrate the taxonomy's value for research and practice, fulfilling the criterion of usefulness.

#### 4.1.4 Parsimoniousness

The final taxonomy comprises seven domains and 28 subdomains, though further condensation into two overarching domains (human and environment) is conceivable. These vast superordinate higher-order domains could complement the existing structure, resembling the organization of the PHK taxonomy. Compared to other frameworks and taxonomies, the proposed PHK taxonomy demonstrates greater parsimony, partially meeting this parsimony criterion.

#### 4.1.5 Replicability

Although implementing an interrater loop to ensure replicability, we cannot completely ensure that replicability is met. Ultimately, the categorization is subjective and can deliver diverging results for different scientists. Nonetheless, this criterion is partially met, as we have used the interrater loop as a safeguard. Additionally, the assignment of the elements to the domains should be comprehensible based on the given definitions. Further, the taxonomy's structure may invite criticism for its emphasis on human-centered domains. Four out of seven domains (*Health, Education, Safety, Standard of Living*) focus directly on individuals, while one (*Political and Economic Systems*) addresses politics and broader societal structures. N*utrition* combines man and environmental concerns, encompassing the ecological impact of agriculture and fisheries. Only one domain (Environment) exclusively addresses environmental issues, potentially creating an imbalance favoring human-centered perspectives. Empirical evidence will show whether reorganization is needed and whether the taxonomy will serve practical purposes effectively.

### 4.2 Mapping further concepts and scientific branches into the field of PHK

Beyond the UN's SDGs, GC frameworks, and the PH framework, numerous other frameworks consider complex human-environment interactions to different extents. The *Planetary Boundaries* (49, 50) are recognized within the PH framework (29) on which the PHK taxonomy is, among others, based. Although we do not directly feature the nine planetary boundaries in the taxonomy, we can map them to the PHK subdomains: climate change, ocean acidification, novel entities, and ozone depletion could be located within the *Environmental Change* subdomain; biosphere integrity within the *Biodiversity s*ubdomain, while freshwater use could be mapped to *Food and Water Management*. This model is too narrow in scope to serve as a blueprint for our taxonomy. However, the Planetary Boundaries are too specific to serve as standalone subdomains in the PHK taxonomy. Instead, they can be effectively integrated across multiple subdomains and facets, enriching its scope.

The *Doughnut model* is a more comprehensive model integrating planetary and societal boundaries (31, 59), many of which can be found within the PHK taxonomy. Health can be directly transferred to *Health* (P1), education to *Education* (P2), and peace and justice to *Safety* (P4). Other social boundaries can be allocated on the level of subdomains: water and food can be located within P2 *Food Management*; Income and work within P6 *Wealth*; networks, housing, and energy within P6 *Possessions*; political voice within P7: *Participation*; and social equity within P7: *Welfare*. As discussed above, gender equality can be found wherever applicable.

The *Planetary Health Literacy* (PHL) framework (1) refers to knowledge and competencies about planetary health for responsible judgment and decision making to enhance health at individual, societal, and planetary levels. While the PHL framework does not specify knowledge domains, it emphasizes the importance of planetary health knowledge and skills. We propose integrating the PHK taxonomy into this framework to fill the gap of unspecified knowledge.

*One Health* is a framework that acknowledges that human, animal, and environmental health are closely interlinked. We do not want to deny this. All components of One *Health* are represented in the PHK taxonomy. Although each element appears separately to create disjunction, interactions between the domains, subdomains, and facets must also be established. Thus, it is important to consider knowledge not only about the components alone but also about their interactions, such as the interactions between human, animal, and environmental health.

*Buen Vivir* and *Degrowth* are other concepts that feature sustainable development from another perspective and different worldviews. To the best of our knowledge, these concepts are not as well formulated and elaborated and do not contain a collection of global issues, so we did not include them in the taxonomy development. Further, we wanted to keep neutral in terms of underlying worldviews. However, viewing such global problems from different perspectives would be useful to gain a comprehensive overview of this field and should be reflected in educational programs or knowledge tests.

### 4.3 Implications of the PHK taxonomy

The literature-based developed PHK taxonomy is a theoretical framework for organizing knowledge elements relating to the environment, human health and human systems. Empirical multivariate research is essential to evaluate its validity, beginning with ensuring comprehensive domain coverage. Methods such as corpus linguistics and large language models could enhance unbiased and exhaustive domain coverage.

A crucial second step is the analysis of the taxonomy's structure in empirical knowledge data. The PHK taxonomy will serve as a blueprint for developing a PHK test with psychometrically robust items that represent all domains of PHK in equal extent. Items best representing the respective domain will be selected in initial piloting of domain-specific item sets, followed by testing with a reduced item pool across domains to investigate the factor structure of PHK.

The PHK taxonomy suggests a higher-order factor structure with a general PHK factor and seven subordinate domains-specific factors. However, although we usually conceptualize declarative knowledge as a construct with a hierarchical structure (60, 61), these statistical models might be just a simplistic abstraction that simplifies the visualization, comprehension, and communication of knowledge (61).

PHK extends beyond psychology as highlighted by its inclusion in recent educational frameworks and the PISA-2022 framework (7). Education on sustainability, the environment, and social issues (implying knowledge) is deemed a critical political and educational challenge. Politicians and researchers see this knowledge a necessary—but insufficient—prerequisite for addressing global issues. A PHK-test could serve as a measure to track individuals' knowledge about the world's major issues and their engagement in global problem-solving. In (intensive) longitudinal studies, tracking people's knowledge, and developing educational interventions to advance the knowledge and associated behaviors would be possible. This requires the development of short- and ultra-short test forms that can be in longitudinal panel surveys, along with parallel test forms ensuring equality considering construct coverage, difficulty, and measurement precision while circumventing test repetition effects (62, 63).

Future research questions will focus validating the PHK test and embedding it into the broader nomological network. A key question is PHK's placement within the broad field of general knowledge. PHK is not the same as, but rather an inherent part of declarative fact knowledge. Recent research identified four broad knowledge areas (natural sciences, life sciences, life sciences, humanities) and several subordinate subdomains reflecting the structure of traditional school curriculums (54). PHK is only partly taught in school and transcends these categories and spans across the borders of traditional school curricula.

For instance, PHK content such as crime, human rights, governance and economy aligns with social sciences (in the domains politics, economy, finances), while health and nutrition fit into life sciences (in between the domains medicine, psychology, nutrition, health), and some environmental issues within natural sciences (in between biology, geography, ecology). Consequently, PHK is likely to correlate more strongly with social, natural, and life sciences than with humanities. Despite its interdisciplinary nature, each aspect of PHK can be mapped somewhere into the field of general knowledge.

Last, the taxonomy's structure, with seven domains and 28 subdomains, offers a useful tool for transferring and communicating knowledge. It enables zooming in and out, facilitating granular exploration of topics and integration into broader contexts. This versatility allows the development of educational material and teaching units suitable for integration into standard curricula and teaching routines or extraordinary teaching units. Integrating PHK into standard curricula would make sense to reach students, regardless of their background, age, and educational level who can learn about the components and their interrelations. Knowledge of the individual components and their interrelationships could be deepened throughout the school years in order to achieve a basic understanding. At the same time, the exact content and teaching material must be adapted to the student's educational level. Educators could use the zoom-in/zoom-out approach, providing a broad overview of PHK or focusing on specific domains for in-depth exploration. Additionally, PHK could be integrated into vocational education, focusing in particular, but not exclusively, on those aspects that are relevant in the specific fields (e.g., health care workers).

## 5 Conclusion

In this contribution, we introduced the Planetary Health Knowledge (PHK) taxonomy that targets knowledge about the health of the planet, the health of humans and human civilization, and their interactions. We developed the taxonomy by merging the highly redundant approaches of Sustainable Development, Global Citizenship, and Planetary Health, indicating the existence of jangle fallacies. The taxonomy is exhaustive, as disjunct as possible, of social relevance, and parsimonious and comprises seven domains: *Health, Nutrition, Environment, Safety, Education, Standard of Living*, and *Political and Economic Systems*. The PHK taxonomy can be used to develop a comprehensive PHK test as well as tests focusing on specific aspects of the taxonomy. Short and parallel forms of a general PHK test will be suitable for investigating the nomological network of PHK and for use in the field of application for longitudinal and intervention studies.

## Data Availability

We provide an alternative vizualisation of the PHK taxonomy at the Open Science Framework. This data can be found at: Open Science Framwork: https://osf.io/ebdkg/?view_only=eec79d0fa0d94dde99819e23f27296d9.

## Footnotes

^1^Rees F, Wilhelm O. *What we know about the world. Development of a planetary health knowledge test*. (Manuscript in preparation).
